# Diverse subtypes of astrocytes and their development during corticogenesis

**DOI:** 10.3389/fnins.2015.00114

**Published:** 2015-04-07

**Authors:** Hidenori Tabata

**Affiliations:** Department of Molecular Neurobiology, Aichi Human Service Center, Institute for Developmental ResearchKasugai, Japan

**Keywords:** astrocyte, oligodendrocyte, cerebral cortex, subventricular zone, gliogenesis, cell specification

## Abstract

Astrocytes are one of the most abundant cell types in the mammalian central nervous system, and are known to have a wide variety of physiological functions, including maintenance of neurons, formation of the blood brain barrier, and regulation of synapse functions. Although the migration and positioning of neurons has been extensively studied over the last several decades and many aspects have been uncovered, the process underlying glial development was largely unknown until recently due to the existence of multiple subtypes of glia and the sustained proliferative ability of these cells through adulthood. To overcome these difficulties, new gene transfer techniques and genetically modified mice were developed, and have been gradually revealing when and how astrocytes develop during corticogenesis. In this paper, I review the diversity of astrocytes and summarize our knowledge about their production and migration.

## Introduction

Astrocytes are among the most abundant types of glia, and the ratio of astrocytes to neurons has been shown increase with primate evolution (Bass et al., 1971). Recent studies have indicated that astrocytes not only provide support to neurons, but also actively regulate the physiological functions of the brains, and that astrocyte dysfunction can lead to developmental and/or psychiatric disorders (Molofsky et al., 2012; Burda and Sofroniew, 2014; Sloan and Barres, 2014). Despite their existence in abundance and their physiological importance, the processes underlying the development of astrocytes are largely unknown. This is partly due to the occurrence of diverse subtypes of astrocytes. The morphologies and functions of these cells differ among sites of the brain and among species. In addition, the cells have multiple origins and their proliferation persists into adult life, making analysis of the fates of these cells more complex. However, the recent introduction of novel techniques, including mice expressing region-specific Cre recombinase and *in utero* electroporation of transposon vectors have helped in revealing, at least in part, the process of normal development of astrocytes in the brain. In this brief review article, I focus on the development of astrocytes in the cerebral cortex. I first summarize the subtypes of astrocytes and their functions in rodents and primates. I then describe the migration of these subtypes from the cortical ventricular zone (VZ), and from other sites. I also describe in brief the process of development of oligodendrocytes, and compare it to that of astrocytes.

## Heterogeneity of astrocytes

The existence of two basic subtypes of astrocytes in rodents, the protoplasmic and fibrous astrocytes, has been established beyond doubt (Miller and Raff, 1984). Protoplasmic astrocytes posses highly branched bushy processes and are widely distributed in the gray matter. They extend endfeet to blood vessels and enwrap them to form the glial limiting membrane, which is the outermost wall of the blood brain barrier (BBB). They are also closely associated with synapses with its processes and play diverse roles, such as clearance of glutamate (Rothstein et al., 1996; Oliet et al., 2001), modulation of synaptic functions (Henneberger et al., 2010; Uwechue et al., 2012), and regulation of local blood flow in response to synaptic activities (Simard et al., 2003; Takano et al., 2005). Protoplasmic astrocytes have also been reported to participate in the formation and elimination of synapses (Pfrieger, 2010; Kucukdereli et al., 2011). Interestingly, the processes of two adjacent protoplasmic astrocytes are mutually exclusive, and occupy non-overlapping domains (Bushong et al., 2002; Ogata and Kosaka, 2002; Halassa et al., 2007). The domain of a single astrocyte covers about 100,000 synapses in mice (Bushong et al., 2002), and these synapses can be simultaneously regulated by one astrocyte as a synaptic island (Halassa et al., 2007).

**Figure 1:**
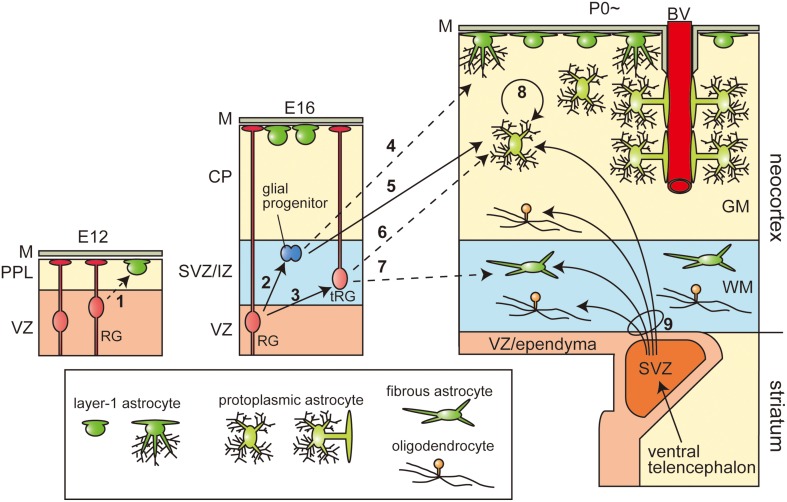
**Heterogeneity of astrocytes and the multiplicity of their origin**. The three pictures represent the production and final positioning of the astrocytes and oligodendrocytes in the developmental stages. The stages in the mice are given above each picture (E, embryonic day; P, postnatal day). Arrows with solid lines indicate the cell lineages confirmed by lineage tracing experiments. Arrows with broken lines show the hypothetic cell lineages by histological investigations, but not confirmed by precise lineage tracing. Neurons and OPCs are not shown. GM, gray matter; WM, white matter; M, meninges or pia matter; PPL, primordial plexiform layer; VZ, ventricular zone; SVZ, (embryonic or postnatal) subventricular zone; IZ, intermediate zone; CP, cortical plate; BV, blood vessel.

On the other hand, fibrous astrocytes possess straight and long processes and are mainly located in the white matter. In this cell type, the expressesion level of glial fibrillary acidic protein (GFAP), an intermediate filament protein, is higher than that in the protoplasmic astrocyte, in which the GFAP protein is sometimes found only in the endfeet on the blood vessels (Oberheim et al., 2009). The functions of fibrous astrocytes are not clear. At least, these cells associate with the blood vessels via their processes just like the protoplasmic astrocytes (Marín-Padilla, 1995). In addition to these basic cell types, there are specialized astrocytes in Layer 1 of the murine cerebral cortex that show a bushy morphology similar to that of protoplasmic astrocytes in the gray matter, but strongly express GFAP like fibrous astrocytes. Their processes cover the outer surface of the brain parenchyma just under the pia matter and form the glial limiting membrane, which continues into the other part of the glial limiting membrane formed by the endfeet of the protoplasmic astrocytes, as described above (Figure 1). GFAP-positive fibroblast-like cells have been reported to exist on the pial surface, (García-Marques and López-Mascaraque, 2013; Martín-López et al., 2013). These cells also cover the outer surface of the brain with their cell bodies to participate in the formation of the glial limiting membrane. Although the subtypes of astrocytes described above, namely fibrous, protoplasmic and Layer-1 astrocytes, are widely found in mammalian brains, there are at least two specific subtypes for human or other primates (Colombo and Reisin, 2004; Oberheim et al., 2009; Sosunov et al., 2014). In Layer 1 of the primate cerebral cortex, there are densely packed GFAP^+^/CD44^+^ astrocytes called interlaminar astrocytes (Colombo and Reisin, 2004). These cells extend straight and poorly branched processes that are about a millimeter long into the cortical gray matter, frequently terminating on the blood vessels in Layers 2–4 (Sosunov et al., 2014). This subtype appears after birth, and in the fetal stages the glial constituents in the Layer 1 are similar to that of rodents, and thus, transformation of Layer-1 astrocytes with short processes to interlaminar astrocytes has been suggested (Marín-Padilla, 1995; Colombo et al., 1997). The second subtype that is primate-specific is the varicose projection astrocytes, which are also GFAP^+^/CD44^+^ and are situated mainly in Layers 5 and 6. This cell type extends many straight 100-μm long processes and one to five up to 1-mm long processes with many varicosities (Oberheim et al., 2009; Sosunov et al., 2014), which may terminate in the neuropil or on the vasculature. In human, protoplasmic and fibrous astrocytes also exhibit unique structure. They have been reported to be 2~2.5-fold larger in diameter in the human cortex than in the mouse (Oberheim et al., 2009). Human protoplasmic astrocytes also form exclusive domains like the cells in rodents, and a single domain covers about 2,000,000 synapses. In the deeper layers of the human cortex, protoplasmic astrocytes and varicose projection astrocytes coexist, and their processes are intermingled, suggesting that they are distinct subtypes of cells with differing functions.

## Glial production in the cortical VZ

Astrocytes in the cerebral cortex are produced from the cortical ventricular zone (VZ) or from the ventral forebrain. In the cortical VZ of mammalian embryonic/fetal brains, there are cells called radial glia (RG), which extend long ascending processes called radial fibers to the pial surface and act as a scaffold for neurons migrating from the VZ toward the pial surface. RG were labeled as “glia” because they show several features of astrocytes, such as glycogen granules (Schmechel and Rakic, 1979; Gressens et al., 1992) and express GFAP, especially in the human fetus (Levitt et al., 1981; Cameron and Rakic, 1991). However, they are actually not differentiated glia, but neural stem cells, which generate neurons during the early to late cortical development, and later, glia (Fujita, 1963; Miyata et al., 2001; Noctor et al., 2001). There is a longstanding debate on whether RG in the cortical VZ are homogeneous and whether their potential changes from neuronal production to glial production during the course of development, or whether the RG population includes neuron-restricted progenitors and glia-restricted progenitors even from the early stage of cortical development and the glial progenitors are in a dormant state until the late stages. Although several lines of evidence support the latter (Levitt et al., 1981; McCarthy et al., 2001), recent lineage tracing experiments using mixed retroviruses (Costa et al., 2009) and the Mosaic Analysis with Double Markers (MADM) technique (Gao et al., 2014) have shown no significant numbers of glia-restricted progenitors in the early stages. Recently, a new glial lineage tracing system using transposon plasmid vectors, which integrates into the host genome in the presence of transposase (Kawakami and Noda, 2004; Sato et al., 2007), has been developed. It has been demonstrated that introduction of the transposon vector together with the transposase expression vector by *in utero* electroporation (Fukuchi-Shimogori, 2001; Saito and Nakatsuji, 2001; Tabata and Nakajima, 2001) successfully labeled glial cells (Yoshida et al., 2010). Using this technique, Siddiqi et al. demonstrated that the RG were first exclusively GLAST^+^/Nestin^+^ and produced neurons preferentially, and then GLAST^+^/Nestin^−^ progenitors emerged within the RG population in the later stages, that preferentially produced astrocytes (Siddiqi et al., 2014), demonstrating a potential shift from neuronal to glial production from RG. Moreover, Noctor et al. directly observed that the neural stem cells first produced neurons by asymmetric cell divisions and then the same cells differentiated into astrocytes in long-term live imaging on slice culture (Noctor et al., 2004). Based on the aforementioned evidence, the former hypothesis is now widely accepted.

After specification of the glial lineage, the glial progenitors migrate into cortical gray matter and white matter and differentiate into protoplasmic and fibrous astrocytes, respectively. The most accepted model of such migration of glial progenitors is the direct transformation of RG (Figure 1, arrow-3, 6, 7), in which the radial fibers are retracted to elevate the cell soma from the VZ. This cell movement is similar to that identified in the neuronal migration process called “somal translocation” (Nadarajah et al., 2001), and the cells under such transformation are called transforming RG (tRG). The morphology of tRG has been observed repeatedly by Golgi staining, immunostaining for GFAP, and carbocyanine dye (DiI) staining (Schmechel and Rakic, 1979; Voigt, 1989; Gressens et al., 1992; deAzevedo et al., 2003). The differentiation of tRG cells into astrocytes has been directly shown by live imaging on slice culture (Noctor et al., 2004). On the other hand, astrocytes are also thought to arise from proliferative glial progenitors in the subventricular zone (SVZ; Figure 1, arrow-2, 4, 5). It would be of interest to know which progenitors produce which subtypes of astrocytes. Gressens et al. administrated [3H]-thymidine to E17 mice, after completion of neurogenesis, and observed that the GFAP- or RC2-positive [3H]-thymidine labeled cells (proliferative glial progenitors) were first found in the SVZ or IZ and gradually shifted toward the pial surface and positioned themselves in the upper cortical plate, but not in the white matter. Moreover, they administrated methylazoxymethanol acetate (MAM), which eliminates proliferative cells, to E17 and E18 mice, and observed the greatly reduced number of protoplasmic astrocytes in the upper cortical plate, with no significant effect on the generation of the fibrous astrocytes in the white matter (Gressens et al., 1992), suggesting that the proliferative glial progenitors in the SVZ only differentiate into protoplasmic astrocytes (Figure 1, arrow-5). On the other hand, Cai et al. demonstrated that postnatal genetic deletion of Olig2, a transcription factor known to be essential for glial differentiation (Ono et al., 2008), resulted in a severe deficit in the formation of fibrous astrocytes, but no significant difference in the number of protoplasmic astrocytes in the upper cortical plate (Cai et al., 2007), indicating that these two classical subtypes are generated in different ways. Recently, the multi-color lineage tracing system for astrocytes, called the “Star Track” method, has been developed by modifying the transposon vector system (García-Marques and López-Mascaraque, 2013; Martín-López et al., 2013). Consistent with the results of the traditional retrovirus lineage tracing experiments (Price and Thurlow, 1988; Levison et al., 1993), Star Track also demonstrated that most of the clones were either exclusively protoplasmic or exclusively fibrous astrocytes, suggesting that these two types of astrocytes are generated from independent progenitors, although it remains unknown as to which progenitors they might be.

The process of generation of Layer-1 astrocytes was also found to be unique. By intensive observations using Golgi staining, Marin-Padilla proposed that the Layer-1 astrocytes are produced in two waves (Marín-Padilla, 1995). In the very early stage of cortical development, the primordial plexiform layer (PPL), which is also called preplate, is formed just outside the VZ. In this stage, a subset of VZ-derived cells move onto the basal lamina underling the pia matter, and differentiated into first Layer-1 astrocytes and form the subpial glial limiting membrane (Figure 1, arrow-1). As development proceeds the population of Layer-1 astrocytes adopts newly generated astrocytes probably derived from the SVZ (Figure 1, arrow 4). It is not clear whether these two different origins of the Layer-1 astrocytes correspond to two types of Layer-1 astrocytes, namely fibroblast-like and protoplasmic-like astrocytes. Nevertheless, the Star Track analyses revealed that the clones of these subtypes of Layer-1 astrocytes are highly exclusive of each other (García-Marques and López-Mascaraque, 2013; Martín-López et al., 2013). It has been reported that a subset of protoplasmic astrocytes arises from the Layer-1 astrocytes or multipotent progenitors in the layer 1 of the cerebral cortex (Marín-Padilla, 1995; Costa et al., 2007).

As the brain increases in size during the first 20 postnatal days in mice, the number of glia increases dramatically (Bandeira et al., 2009). However, direct transformation of RG may produce a limited number of astrocytes, and the production of astrocytes from the proliferative glial progenitors in the SVZ almost ends by P14 (Levison et al., 1993), suggesting the additional cell-amplifying system. By using two-photon microscopy, Ge et al. observed the frequent cell divisions of the protoplasmic astrocytes in P5 hGFAP-GFP mice with an open skull, but an intact pial surface (Ge et al., 2012) (Figure 1, arrow-8). The dividing cells were not migrating glial progenitors, but differentiated protoplasmic astrocytes settled in the cortical gray matter. These dividing astrocytes extended highly branched processes, contacting the blood vessels with their endfeet, and coupled with surrounding mature astrocytes with gap junctions. This local production was estimated as the major source of protoplasmic astrocytes in the adult brain.

## Multiple origins of glia

Glia of the cerebral cortex are also produced from the postnatal SVZ, a specialized reservoir of glial and neuronal progenitors. The postnatal SVZ is represented by the wedge-shaped structure between the pallium and subpallium, and is composed of Zebrin II (aldolase C)-positive cortical VZ-derived cells, mainly located in the periphery, and Dlx2-positive ventral telencephalon-derived cells populating the center (Marshall and Goldman, 2002). Lineage tracing after direct injection of a retrovirus into the postnatal SVZ revealed that while the neurons migrated anteriorly to the olfactory bulb and differentiated into granular and periglomerular interneurons (Alvarez-Buylla and Garcia-Verdugo, 2002), the glial progenitors migrated dorsally and differentiated into both astrocytes and oligodenderocytes in the gray and white matter (Levison and Goldman, 1993; Parnavelas, 1999; Marshall and Goldman, 2002) (Figure 1, arrow-9). The proportions of astrocytes and oligodendrocytes produced from this structure show temporal changes. The glial progenitors of the P2 SVZ in the neonatal rat gave rise to astrocytes mostly in the cortical gray matter, while the P14 SVZ cells mainly differentiated into oligodendrocytes in the white matter (Levison et al., 1993). Within the MGE-derived cell population, Olig2 acts as a determinant of the glial fate. Overexpression of wild-type Olig2 using retrovirus increased the production of both astrocytes and oligodendrocytes, while overexpression of the dominant-negative form of Olig2 increased the production of neurons (Marshall et al., 2005).

As another possible source of astrocytes, oligodendrocyte progenitors (OPCs) cannot be ignored. OPCs express several specific markers, such as NG2 and platelet-derived growth factor receptor α (PDGFRA), and are distributed widely in the late embryonic and postnatal brains. OPCs collected from the rat optic nerve using A2B5 mononclonal antibody, which binds to an early OPC-specific ganglioside (Eisenbarth et al., 1979; Schnitzer and Schachner, 1982; Raff et al., 1983a), were shown to differentiate into GFAP^+^ astrocytes in culture in the presence of serum factors (Raff et al., 1983b). The resulting astrocytes from the OPCs in culture are called type 2 astrocytes, while those from the cortical VZ are called type 1 astrocytes, because they exhibit different morphologies. The ability of cultured OPCs to produce astrocytes *in vivo* was shown by transplantation. When human A2B5^+^, PSA-NCAM^−^ cells taken from 17- to 23-week forebrains were expanded in culture with fetal bovine serum and grafted into newborn mice at P0 or P1, they gave rise to astrocytes as well as NG2 cells and oligodendrocytes (Windrem et al., 2004, 2008, 2014; Han et al., 2013). These observations indicate the potential of OPCs to produce astrocytes. However, differentiation of OPCs into astrocytes during the course of normal development of brains seems minor, if any. When OPCs were cultured in serum-free medium and grafted into P5 rats, OPCs differentiated only into oligodendrocytes but not astrocytes (de los Monteros et al., 1993). Moreover, lineage tracing experiments using transgenic mice that express Cre recombinase in the OPCs (NG2-Cre and PDGFRA-CreERT2) revealed that OPCs produce oligodendrocytes in the gray and white matter, but not astrocytes in the neocortex, although some astrocytes were produced in the ventral forebrain (Zhu et al., 2007, 2012; Rivers et al., 2008).

As described above, astrocytes in the cerebral cortex have multiple origins, and are functionally and morphologically diverse. This raised the question of whether the progenitors at different sites of the brain produce functionally identical populations of astrocytes and compensate cell numbers, or produce different subtypes of astrocytes. The results of an experiment using the Cre-loxP lineage tracing system showed that oligodendrocytes in the cerebral cortex are also produced from different sites depending on the developmental stages (Kessaris et al., 2006). The first wave of production begins around E12.5 from Nkx2.1-expressing precursors in the MGE and anterior entopeduncular area (AEP). The second wave begins around E15 from Gsh2-expressing LGE and the caudal ganglionic eminence (CGE), and finally, local production begins in the Emx1-expressing cortical VZ around birth. When any one of these production sites is eliminated by expressing diphtheria toxin A fragment (DTA) under the control of the same Cre driver mouse lines, the OPCs from the other sites cover the deficient area (Kessaris et al., 2006). Furthermore, the oligodendrocyte lineage cells derived from the Nkx2.1-progenitors decreased during postnatal life, and were replaced with newly generated Gsh2- and Emx1-derived cells. Hence, oligodendrocytes derived from three different progenitor domains are functionally replaceable by each other, and compete to populate the limiting space in the cerebral cortex. This situation is referred to as “oligodendrocyte wars” (Richardson et al., 2006). However, this is not the case for astrocytes, especially in the spinal cord. Astrocytes in the spinal cord are produced from different progenitor domains arrayed in a dorsal to ventral pattern in the VZ. When one of the progenitor domains is eliminated by specific expression of DTA, neighboring astrocytes or their progenitors do not enter the deficient area to cover the functions (Tsai et al., 2012). In the cerebral hemispheres, however, substantial amounts of glial progenitors migrate from the MGE and differentiate into astrocytes as mentioned above. In fact, the astrocytes derived from Dlx2-expressing progenitors in the postnatal SVZ were reported to extend their endfeet onto blood vessels (Marshall and Goldman, 2002), indicating that they are functionally equivalent to the cortical VZ-derived astrocytes. Moreover, in the transplantation experiments of A2B5^+^/PSA-NCAM^−^ human glia progenitors, astrocytes in the host mouse brains were gradually replaced by human astrocytes derived from the donor cells (Han et al., 2013; Windrem et al., 2014), suggesting cell-cell competition among the astrocytes for their exclusive domains in the limited space of the cerebral hemispheres. This situation should be called “astrocyte wars.” Interestingly, the implanted human glial progenitors developed in a cell-autonomous manner in the host mouse brains, and generated protoplasmic astrocytes of larger diameter than the host cells and also varicose projection astrocytes having several long unbranched processes with many varicosities. Surprisingly, the resulting humanized chimeric mice represented higher LTP and higher learning ability than the control mice (Han et al., 2013), suggesting that the higher intellectual activity of humans is, at least a part, due to the human-type astrocytes.

## Perspectives

In this article, I have described the heterogeneity of astrocytes among different sites of the cerebral cortex and among different animal species. I have also described the several distinct origins of astrocytes. As at present, the relationships between the origins and subtypes of astrocytes are not yet fully clarified. For example, the development and specification of protoplasmic and fibrous astrocytes are still not clear, even though they are the most basic subtypes of astrocytes. Recent studies have revealed many aspects of the physiological importance of astrocytes, such as the regulation of synapse functions and blood flow, which has drawn a lot of attention to the process of glial development, maturation and plasticity. Moreover, novel methods of lineage tracing and gene transfer for glial progenitors have been developed using transposon or Cre-loxP systems, and these modern techniques are now greatly accelerating the accumulation of knowledge in this field. Interestingly, many glia-specific genes have been identified as genes related to developmental and/or psychiatric disorders. To understand the mechanisms underlying the development of these diseases and to develop new clinical treatments, further knowledge of glial development is important.

### Conflict of interest statement

The author declares that the research was conducted in the absence of any commercial or financial relationships that could be construed as a potential conflict of interest.
